# An Exploratory Descriptive Study of Antimicrobial Resistance Patterns of *Staphylococcus* Spp. Isolated from Horses Presented at a Veterinary Teaching Hospital

**DOI:** 10.1186/s12917-017-1196-z

**Published:** 2017-08-22

**Authors:** James Wabwire Oguttu, Daniel Nenene Qekwana, Agricola Odoi

**Affiliations:** 1 Department of Agriculture and Animal Health, College of Agriculture and Environmental Sciences, University of South Africa, Florida Science Campus, Johannesburg, South Africa; 2 Section Veterinary Public Health, Department of Paraclinical Sciences, Faculty of Veterinary Sciences, University of Pretoria, Pretoria, South Africa; 3 Biomedical and Diagnostic Sciences, College of Veterinary Medicine, University of Tennessee, Knoxville, USA

**Keywords:** Horse, *Staphylococcus aureus*, *Staphylococcus pseudintemedius*, *Staphylococcus epidermidis*, *Staphylococcus chromogens*, Antimicrobial resistance, Multi-drug resistance, MDR, Gauteng, South Africa

## Abstract

**Background:**

Antimicrobial resistant *Staphylococcus* are becoming increasingly important in horses because of the zoonotic nature of the pathogens and the associated risks to caregivers and owners. Knowledge of the burden and their antimicrobial resistance patterns are important to inform control strategies. This study is an exploratory descriptive investigation of the burden and antimicrobial drug resistance patterns of *Staphylococcus* isolates from horses presented at a veterinary teaching hospital in South Africa.

**Methods:**

Retrospective laboratory clinical records of 1027 horses presented at the University of Pretoria veterinary teaching hospital between 2007 and 2012 were included in the study. Crude and factor-specific percentages of *Staphylococcus* positive samples, antimicrobial resistant (AMR) and multidrug resistant (MDR) isolates were computed and compared across *Staphylococcus* spp., geographic locations, seasons, years, breed and sex using Chi-square and Fisher’s exact tests.

**Results:**

Of the 1027 processed clinical samples, 12.0% were *Staphylococcus* positive. The majority of the isolates were *S. aureus* (41.5%) followed by *S. pseudintermedius* (14.6%). Fifty-two percent of the *Staphylococcus* positive isolates were AMR while 28.5% were MDR. Significant (*p* < 0.05) differences in the percentage of samples with isolates that were AMR or MDR was observed across seasons, horse breeds and *Staphylococcus* spp. Summer season had the highest (64.3%) and autumn the lowest (29.6%) percentages of AMR isolates. Highest percentage of AMR samples were observed among the Boerperds (85.7%) followed by the American saddler (75%) and the European warm blood (73.9%). Significantly (*p* < 0.001) more *S. aureus* isolates (72.5%) were AMR than *S. pseudintermedius* isolates (38.9%). Similarly, significantly (*p* < 0.001) more *S. aureus* (52.9%) exhibited MDR than *S. pseudintermedius* (16.7%). The highest levels of AMR were towards β-lactams (84.5%) followed by trimethoprim/sulfamethoxazole (folate pathway inhibitors) (60.9%) while the lowest levels of resistance were towards amikacin (14.%).

**Conclusions:**

This exploratory study provides useful information to guide future studies that will be critical for guiding treatment decisions and control efforts. There is a need to implement appropriate infection control, and judicious use of antimicrobials to arrest development of antimicrobial resistance. A better understanding of the status of the problem is a first step towards that goal.

## Background

*Staphylococcus* are Gram-positive cocci that comprise of over 50 species and subspecies, some of which are common commensals of various body sites of different animals [1, 2]. Although many *Staphylococcus* spp., are of no clinical significance, some are important opportunistic pathogens [1, 3, 4]. In equine medicine *S. aureus*, *S. intermedius* and *S. hyicus*, have been associated with clinical infections [2]. From a public health point of view, there are increasing reports of highly virulent staphylococcal infections that can be transmitted between horses and humans [5–7]. For example, a Canadian study reported that 96% of the *Staphylococcus* samples from horses and 93% from humans displayed similar genetic profiles [8]. Another study done in the Netherlands reported that *S. aureus* isolated from a 16 year old girl was genetically similar to that isolated from a horse [5]. Busscher et al. [3] also reported identical Methicillin Resistant *Staphylococcus aureus* (MRSA) from horses and their caregivers.

Infections with antimicrobial drug resistant *Staphylococcus* spp. in both equine and human medicine has been associated with high morbidity, mortality and treatment costs. In animals, infections with antimicrobial drug resistant *Staphylococcus* spp. has also been associated with significant animal welfare implications due to animals staying sick for long periods in event of treatment failures [9–12]. Although coagulase-positive staphylococci (CoPS) are the most important groups associated with severe infections, coagulase-negative staphylococci (CoNS) have emerged as important pathogens as well. Moreover, all *Staphylococcus* spp., regardless of their coagulase activity, have potential to develop resistance to different classes of antimicrobials used for human and animal treatment [13]. Just like CoPS, resistance to antimicrobials such as gentamycin, macrolides, tetracycline, streptomycin, trimethoprim, sulfamethoxazole and fluoroquinolones is commonly observed among CoNS isolates from pets and horses [13, 14].

Excessive use of broad spectrum antimicrobials has been hypothesized as the main driver of antimicrobial drug resistance in *Staphylococcus* spp. [15]. For example, Bagcigil et al. [16] reported high levels of resistance to multiple antimicrobial agents including β-lactams in horses with previous history of treatment with β-lactams. Failure to complete the course of antimicrobial treatment has also been identified as a risk factor for development of resistance among staphylococcal isolates from horses [17]. Recent studies show that colonization with *Staphylococcus* spp. carrying antimicrobial resistance genes increases the risk of infection with resistant *Staphylococcus* [2, 17]. Transfer of resistance genes between pathogenic organisms and commensal flora has also been hypothesized as a risk factor for infection with resistant isolates [18]. Morton et al. [19] were able to demonstrate horizontal transfer of one conjugate mupirocin plasmid by finding the same plasmid in different staphylococcal isolates from patients in different areas of the hospital, which suggested that isolates had acquired new genes. Moreover, conjugative plasmids can move between CoPS and CoNS [19]. Furthermore, molecular epidemiological analyses by pulsed-field gel electrophoresis has shown that horizontal transfer of plasmid borne genes within and between different equine staphylococcal species is possible [20].

Despite the existence of evidence suggesting that horses play a significant role as sources of staphylococcal infections for humans [5, 6], it is surprising that studies of staphylococcal infections in Africa in general, and South Africa in particular, have mainly focussed on humans [12, 21, 22]. Available studies in human medicine suggest a high prevalence of resistant staphylococcal infections, especially MRSA, associated with both hospital and community acquired infections [9]. Therefore, studies of horses are critically needed to fill this knowledge gap. Given the paucity of data on both the burden of staphylococcal infections and the antibiotic resistance profiles among *Staphylococcus* isolates from horses in Africa, the present study is an exploratory descriptive investigation of the burden of staphylococcal infections and their antimicrobial drug resistance patterns among horses presented at the University of Pretoria veterinary teaching hospital from 2007 to 2012. This exploratory investigation is expected to provide useful information to guide future hypothesis driven studies in this region of the world.

## Methods

### Study area

This study was conducted using retrospective laboratory data collected from Gauteng province in South Africa. Gauteng province is approximately 18,178 km^2^ in size, and has an estimated population of 13.2 million people (24% of the South African population). During the time period covered by this study, the province had a total of five metropolitan municipalities: Ekurhuleni, Sedibeng, Johannesburg, Tshwane and West Rand. The province has a subtropical climate, and is cooler in Johannesburg and slightly warmer in Pretoria. Gauteng is located in the Highveld region of South Africa, and has an annual summer rainfall of approximately 700 mm. It has four seasons: summer (November–March), autumn (April–May), winter (June–August) and spring (September–October). Winter is the driest season, while December and January are the wettest months. The province experiences annual maximum temperatures of about 22 °C in the south and 25 °C in the north [23, 24].

### Data source

Laboratory records of all 1027 clinical samples from horses, from Gauteng Province, presented at the University of Pretoria bacteriology diagnostic laboratory for isolation and susceptibility testing of *Staphylococcus* spp. from January 2007 to December 2012 were included in the study. The data were received as paper records, reviewed and entered into an electronic database. The following fields were extracted for each record: horse breed, sex, age (in months), date sample was submitted as well as culture and antimicrobial susceptibility test results. The data were assessed for duplicate entries and if any horses had been sampled multiple times during the study period. No duplicates were identified. The dataset did not contain multiple tests from the same horse, neither were there mixed infections in the samples analysed. The breeds of horses were re-classified to identify the top 10 breeds. All other breeds that had small numbers were grouped into one category and called other breeds.

### Isolation of *Staphylococcus* spp. and testing for antimicrobial susceptibility

The Bacteriology Laboratory at the University of Pretoria veterinary teaching hospital, from where the study data were obtained, follows standardized protocols for isolation of *Staphylococcus* spp. based on the methods described in Quinn et al. [25]. Susceptibility testing of samples was conducted using the Kirby Bauer disc diffusion technique following the guidelines described in the Clinical and Laboratory Standards Institute (CLSI) document [26]. The isolates were subjected to antimicrobial susceptibility testing against a panel of 9 drugs using the disc diffusion method. Antimicrobial resistance (AMR) was defined as resistance to at least one antimicrobial while multidrug resistance (MDR) was defined as resistance to 3 or more classes of antimicrobials [27]. Thus, some of the isolates classified as AMR were also included in the MDR group if they exhibited resistance to 3 or more classes of antimicrobials.

### Statistical analysis

Shapiro-Wilks test [28] was used to test normality of continuous variables. Non-normally distributed continuous variables were summarized using medians and interquartile ranges. Age was categorized into four categories: foals (<1 year old), yearlings (1–2 years old), fillies and colts (2–4 years old) and adults (>4 years old). Crude and factor-specific percentages of *Staphylococcus* positive samples, AMR and MDR isolates as well as their 95% confidence intervals were computed. The factors (categorical variables) considered were: species of *Staphylococcus*, geographic location, season, year, breed, age group and sex. Associations between these categorical variables and percentages of *Staphylococcus* positive samples, AMR and MDR isolates were assessed using Chi-square and, in cases of small expected cell sizes, Fishers Exact test. Significance was assessed at *p* ≤ 0.05. Due to the small sample sizes involved in this exploratory descriptive investigation, adjusted associations using multiple regression models could not be assessed. All statistical analyses were performed in STATA [29].

## Results

A total of 1027 clinical samples from horses in Gauteng Province were submitted to the University of Pretoria bacteriology diagnostic laboratory from 2007 to 2012 and were included in this study. Of the 1027 processed samples, 12.0% (123/1027) were positive for staphylococci, the majority of which were *S. aureus* (41.5%) followed by *S. pseudintermedius* (14.6%), *S. epidermidis* (4.9%), *S. equinus* (0.8%), and *S. chromogens* (0.8%). The remaining 37.4% of the samples did not have species information. Of the *Staphylococcus* positive samples, a total of 52.0% (64/123) had isolates that exhibited antimicrobial resistance to at least one antimicrobial (AMR), while 28.5% (35/123) had isolates that exhibited multidrug resistance (MDR). Significantly (*p* = 0.002) more samples had isolates that exhibited AMR (52.0%; 95% CI: 42.8–61.1) than those that had MDR (28.5%; 95% CI: 20.7–37.3%).

### Distribution by animal characteristics

The samples that were submitted and processed, came from 29 different breeds of horses. The five most common breeds contributing samples were: Thoroughbreds (26.8%), European warm blood (12.6%), Arab (11.6%), South African warm blood (6.4%) and Friesian (6.0%) (Table 1). A significant (*p* = 0.001) association was observed between breed and the percentage of *Staphylococcus* positive samples, with the highest percentage of *Staphylococcus* positive samples observed among cross-breeds (20.5%), followed by Boerperds (18.9%) and European breeds (17.8%). There was also a significant (*p* = 0.015) association between breed of the horse and the percentage of samples carrying resistant isolates, with the highest percentage of samples with resistant isolates being observed among the Boerperds (85.7%) followed closely by the American saddler (75%) and the European warm blood (73.9%). Similarly, there was a significant (*p* = 0.006) association between the percentage of samples carrying MDR isolates and horse breed, with the American Saddler having the highest percentage (75%) of MDR followed by European warm blood (56.5%), Thoroughbreds (33.3%) and the Boerperds (28.6%).

**Table 1:** Host factor distribution of equine samples from Gauteng Province (South Africa) tested for *Staphylococcus*, 2007–2012

	All Samples Processed (*n* = 1027)			Staphylococcus Positive Samples (*n* = 123)		
Breed	n^a^	%^b^	95% CI^c^	n^a^	%^b^	95% CI^c^
American Saddler	39	3.8	2.8, 5.2	4	10.3	3.8, 24.6
Arab	119	11.6	9.8, 13.7	8	6.7	3.4, 12.9
Boerperd	37	3.6	2.6, 4.9	7	18.9	9.2, 35.0
Crossbreed	39	3.8	2.8, 5.2	8	20.5	10.5, 36.2
European warm blood	129	12.6	10.7, 14.7	23	17.8	12.1, 25.4
Friesian	62	6.0	4.7, 7.7	2	3.2	0.8, 12.1
Nooitgedachtpony	43	4.2	3.1, 5.6	5	11.6	4.9, 25.3
South African warm blood	66	6.4	5.1, 8.1	1	1.5	0.2, 10.1
Thoroughbred	275	26.8	24.2, 29.6	21	7.6	5.0, 11.4
Welshpony	25	2.4	1.6, 3.6	3	12.0	3.8, 31.9
Percheron	13	1.3	0.7, 2.2	0	0	-
All other breeds	73	7.1	5.6, 8.9	6	8.2	3.1, 17.1
Unspecified or missing	107	10.4	8.7, 12.4	35	32.7	24.0, 42.5
Age Group						
Foal (< 1 year)	278	27.1	24.4, 29.9	58	20.9	16.2, 26.1
Yearling (1–2 years)	83	8.1	6.5, 9.9	9	10.8	5.1, 19.6
Filly or Colt (2–4 years)	146	14.2	12.1, 16.5	13	8.9	4.8, 14.7
Adult (> 4 years)	520	50.6	47.5, 53.7	43	8.3	6.0, 11.0
Sex						
Males	499	48.6	45.5, 51.7	48	9.6	7.2, 12.6
Females	434	42.2	39.2, 45.3	44	10.1	7.6, 13.4
Unspecified or missing	94	9.2	7.5, 11.1	31	33.0	23.6, 43.4

^a^number of samples

^b^percentage of samples

^c^95% confidence interval

The median age of the horses was 4.3 years, but ranged from 0 to 25.7 years (interquartile range: 0.7–9 years). The majority of the samples were from adults (50.6%), followed by foals (27.1%). Yearlings contributed the lowest percentage of samples (8.1%) (Table 1). A significant (*p* = 0.001) association was observed between age group and percentage of *Staphylococcus* positive samples. Foals had the highest percentage (20.9%; 95% CI: 16.2, 26.1%) of *Staphylococcus* positive samples followed by yearlings (10.8%; 95% CI: 5.1, 19.6), fillies/colts (8.9%; 95% CI: 4.8, 14.7%) and adults (8.3%; 95% CI: 6.0, 11.0%). However, there was no significant difference (*p* = 0.360) in the levels of antimicrobial resistance across age groups. On the contrary, a significant (*p* = 0.012) association in the levels of samples that had MDR isolates was observed across age groups, with fillies/colts having the highest levels (46.2%; 95% CI: 19.2, 74.9%) followed by adults (42.0%; 95% CI: 27.0, 57.9%), foals (17.2%; 95% CI: 8.6, 29.4%) and yearlings had the lowest (11.1%; 95% CI: 0.2, 48.2%).

Significantly (*p* = 0.001) more samples from males (48.6%) than from females (42.2%) were processed. However, there were no significant (*p* = 0.438) differences in the percentages of *Staphylococcus* positive samples obtained from males (9.6%) and females (10.1%) (Table 1). Similarly, no significant (*p* = 0.231) differences in the percentage of samples with resistant isolates were observed between males (64.6%); 95% CI: 49.5, 77.8%) and females (52.3%; 95% CI: 36.7, 67.5%). Interestingly, there was a significantly (*p* = 0.024) higher percentage of MDR isolates among samples from male (41.7%; 95% CI: 27.6, 56.8) than female horses (20.5%; 95% CI: 9.8, 35.3%).

### Temporal patterns

The largest percentage of samples were submitted during summer (40.8%) while the lowest (15.6%) were submitted during the spring (Table 2). Although spring (14.4%) and autumn (14.1%) tended to have higher percentages of *Staphylococcus* positive samples than the other two seasons, there was no significant (*p* = 0.357) association between season and percentage of *Staphylococcus* positive samples (Table 2). However, a significant (*p* = 0.002) association between season and percentage of isolates resistant to at least one antimicrobial was observed. Summer had the highest (64.3%; 95% CI: 48.6, 77.4%) and autumn the lowest (29.6%; 95% CI: 15.2, 49.6%) percentage of antimicrobial resistant isolates (Table 2). On the contrary, there was no significant (*p* = 0.137) association between the percentage of MDR isolates and season.

**Table 2:** Temporal and Geographic distribution of equine samples from Gauteng Province assessed for antimicrobial susceptibility, 2007–2012

	All Samples Processed (*n* = 1027)		*Staphylococcus* positive samples (*n* = 123)		AMR^c^ Isolates (*n* = 64)		MDR^d^ *Isolates* (*n* = 35)	
	%^b^	95% CI^a^	%	95% CI^a^	%	95% CI^a^	%	95% CI^a^
Season								
Summer	40.8	37.8, 43.8	10.0	7.4, 13.3	64.3	48.6, 77.4	38.1	24.6, 53.8
Autumn	18.6	16.3, 21.1	14.1	9.9, 19.9	29.6	15.2, 49.6	14.8	5.5, 34.2
Winter	25.0	22.5, 27.8	12.1	8.6, 16.7	58.1	40.0, 74.2	41.9	25.8, 60.0
Spring	15.6	13.5, 17.9	14.4	9.7, 20.7	47.8	28.3, 68.1	8.7	2.1, 29.9
Year								
2007	34.1	31.2, 37.0	7.7	5.3, 11.0	55.6	36.4, 73.2	14.8	5.5, 34.2
2008	15.5	13.4, 17.8	8.2	4.8, 13.6	53.8	27.0, 78.6	15.4	3.6, 47.0
2009	16.1	13.9, 18.4	12.1	7.9, 18.1	55.0	32.9, 75.3	35.0	17.2, 58.3
2010	8.8	7.2, 10.7	13.3	7.7., 22.1	33.3	12.4, 63.9	8.3	1.0, 44.1
2011	7.1	5.7, 8.9	6.8	2.9, 15.5	60.0	16.6, 91.9	20.0	2.1, 74.8
2012	18.5	16.2, 21.0	24.2	18.6, 30.8	52.2	37.7, 66.3	43.5	29.8, 58.2
Municipality								
Johannesburg	20.0	17.6, 22.5	11.7	7.6, 16.9	54.2	34.0, 73.0	33.3	17.2, 54.5
Tshwane	61.2	58.2, 64.2	11.6	9.2, 14.4	49.3	37.9, 60.8	26.0	16.5, 37.6
Ekurhuleni	10.1	8.4, 12.1	10.6	5.4, 18.1	63.6	32.3, 86.5	36.4	13.5, 67.7
Sedibeng	2.4	1.6, 3.6	20.0	6.8, 40.7	40.0	5.3, 85.3	20.0	0.5, 71.6
West Rand	6.2	4.9, 7.9	10.0	7.8, 26.9	60.0	26.3,87.8	30.0	6.7, 65.2

^a^95% confidence interval

^b^Percent

^c^AMR: Antimicrobial resistant (defined as resistance to at least one antimicrobial)

^d^MDR: Multidrug resistant (defined as resistance to at least three classes of antimicrobials; includes a subset of AMR isolates)

A comparison of the percentage of samples submitted and processed each year revealed that significantly more samples were processed in 2007; and the lowest percentage of samples were received and processed in 2011. Moreover, there was a significant (*p* < 0.001) association between year and the percentage of *Staphylococcus* positive samples, with the largest percentage of positive samples occurring in 2012 (24.2%) (Table 2). Thus, except for the anomaly observed in 2011, there was an increasing trend in the percentage of *Staphylococcus* positive samples during the study period (Table 2). By contrast, there were no significant (*p* = 0.707) differences in the percentage of AMR isolates across the years, neither were there significant differences (*p* = 0.304) in the percentage of MDR isolates across the years.

### Geographic patterns

The geographic distribution of sample submissions was a reflection of the distribution of horse populations across the different municipalities with Tshwane submitting most (61.2%) of the samples followed by Johannesburg (20%) (Table 2). Although the percentage of *Staphylococcus* positive samples varied from 10% in Ekurhuleni municipality to 20% in Sedibeng municipality (Table 2), these differences were not statistically significant (*p* = 0.563). The percentage of isolates that were resistant to at least one antimicrobial varied from 40% in Sedibeng to 63.6% in Ekurhuleni (Table 2). Again, there were no significant (*p* = 0.857) differences in the percentage of isolates that were resistant to at least one antimicrobial. Similarly, the percentage of MDR isolates varied from 20% in Sedibeng to 36.4% in Ekurhuleni municipality (Table 2). As was the case with AMR isolates, there were no significant (*p* = 0.891) differences in the percentage of MDR isolates across the municipalities.

### Antimicrobial susceptibility profiles

There was a significant (*p* < 0.001) difference in the percentages of samples with resistant isolates across the *Staphylococcus* spp., with *S. aureus* (72.5%) showing much higher levels of resistance than *S. pseudintermedius* (38.9%) (Table 3). Similarly, there was a significant (*p* = 0.0001) difference in the percentages of samples with MDR isolates across species, and again *S. aureus* (52.9%) exhibited higher levels of MDR compared to *S. pseudintermedius* (16.7%) (Table 3).

**Table 3:** Distribution of antimicrobial resistance by species of *Staphylococcus* and antimicrobial agent

	Resistant Isolates		MDR Isolates	
	%	95% CI	%	95% CI
Species		*n* = 123		*n* = 35
*S. aureus*	72.5	58.3, 83.3^a^	52.9	38.5, 67.1^a^
*S. pseudintermedius*	38.9	19.2, 63.0^b^	16.7	3.6, 41.4^b^
*S. spp* ^*1*^	30.4	18.7, 45.4^b^	2.2	0.1, 11.5^c^
All other species^*2*^	75.0	34.9, 96.8^a^	50.0	15.7, 84.3^a^
Antimicrobial & Antimicrobial Class		*n* = 64		*n* = 35
Aminoglycosides^3^				
Gentamycin	54.6	42.1, 66.7^a^	48.9	38.1, 59.8
Amikacin	14.1	7.3, 25.2^e^	9.1	4.0, 17.1
Β-lactams^4^				
Ampicillin	84.5	72.3, 91.9^b^	37.5	27.4, 48.5
Penicillin	74.1	61.0, 84.0^b^	37.5	27.4, 48.5
Ceftriaxone	44.8	32.3, 58.1^c^	30.0	20.3, 41.3
Fluoroquinolones^5^				
Enrofloxacin	23.4	14.5, 35.7^c^	18.8	10.1, 30.5^b^
Folate Pathway Inhibitors^6^				
Trimotherim-sulfamethoxazole	60.9	48.2, 72.3^a^	53.1	40.2, 65.7^a^
Tetracyclines^7^				
Doxycyline	46.9	34.8, 59.4^a,d^	35.9	24.3, 48.9^c^
Phenicols				
Chloramphenicol	44.1	31.7, 57.2^d^	31.3	20.2, 44.1^c^

^1^Samples that were not identified to species level and were thus reported as *Staphylococcus spp.*

^2^Other species included *S. equinus*, *S. chromogens* and *S. epidermidis*

^3^Gentamycin and amikacin

^4^Penicillin, ampicillin, ceftriaxone

^5^Enrofloxacin

^6^Sulphamethoxazole

^7^Chloramphenicol

^a-d^Estimates with different superscripts are significantly different at 5% significance level

Significant (*p* < 0.05) differences in the levels of resistance to each antimicrobial was also observed, with resistance to β-lactams being the highest (ampicillin: 84.5%; penicillin: 74.1%) followed by Trimethoprim/sulfamethoxazole (folate pathway inhibitors) (60.9%), while the lowest level of resistance was towards amikacin (14.1%) (Table 3). Most MDR isolates tended to exhibit resistance to trimethoprim/sulfamethoxazole (53.1%), followed by gentamicin (48.9%) (Table 3). Only 9.1% of these isolates exhibited resistance towards amikacin and only 18.8% displayed resistance towards enrofloxacin. The distribution of the number of classes of antimicrobials to which isolates were resistant is shown in Table 4.

**Table 4:** Distribution of number of antimicrobial classes to which equine samples exhibited resistance (*n* = 64)

Number of antimicrobial classes to which isolates exhibited resistance	Number of isolates	Percent	95% CI^a^
1	17	26.6	16.3, 39.1
2	12	18.8	10.1, 30.4
3	7	10.9	4.5, 21.2
4	12	18.8	10.1, 30.4
5	8	12.5	5.6, 23.2
6	8	12.5	5.6, 23.2

^a^95% confidence interval

The majority of MDR combinations observed involved *S. aureus* (Table 5). Multidrug resistance involving combinations of four antimicrobials tended to occur more frequently in young foals as compared to combinations that involved more antimicrobials. Most samples with MDR isolates came from Tshwane municipality (Table 5) and involved mainly European warm blood (31.1%) and Thoroughbreds (20.0%). It is interesting to note that a high percentage (35.6%) of MDR isolates were from horses less than 1 month old (Table 5).

**Table 5:** Antimicrobial resistance patterns of staphylococcal isolates from equine samples from Gauteng Province (South Africa), 2007–2012

Horse Age (months)	Breed of Horse	Municipality	Year	Staphylococcus Species	Antimicrobial Resistance Patterns ^a^
0	Thoroughbred	Tshwane	2008	*S. aureus*	AMP-DOX-PEN
0	Europeanwarmblood	Mogale City	2011	*Staph. spp.*	AMP-PEN-SUL
132	Crossbreed	Tshwane	2007	*S. aureus*	AMI-AMP-PEN
0	Boerperd	Tshwane	2007	*S.pseudintermedius*	AMP-DOX-PEN
0	Boerperd	Tshwane	2007	*S.aureus*	AMP-DOX-PEN
0	Unpecified or Missing	Tshwane	2007	*Staph. spp.*	AMP-CEF-PEN
131	Europeanwarmblood	Johannesburg	2008	*S. aureus*	AMP-PEN-SUL
48	Europeanwarmblood	Tshwane	2012	*S.aureus*	AMP-GEN-PEN-SUL
182	Boerperd	Tshwane	2012	*S.aureus*	AMP-GEN-PEN-SUL
1	Arab	Tshwane	2007	*S. equinus*	AMP-CEF-PEN-SUL
96	All other breeds	Tshwane	2012	*S. epidermidis*	DOX-ENR-GEN-SUL
40	All other breeds	Tshwane	2007	*S. aureus*	AMP-CEF-GEN-PEN-SUL
96	Europeanwarmblood	Tshwane	2012	*S. epidermidis*	DOX-ENR-GEN-PEN-SUL
0	Unpecified or Missing	Tshwane	2012	*S. aureus*	AMP-CEF-CHL-PEN-SUL
83	Europeanwarmblood	Johannesburg	2012	*S. aureus*	AMP-CHL-GEN-PEN-SUL
48	Europeanwarmblood	Tshwane	2012	*S. aureus*	AMP-DOX-GEN-PEN-SUL
84	Thoroughbred	Tshwane	2012	*S. aureus*	AMP-CEF-GEN-PEN-SUL
0	Europeanwarmblood	Johannesburg	2008	*S. aureus*	AMP-CEF-GEN-PEN-SUL
0	Unpecified or Missing	Tshwane	2012	*S. aureus*	AMP-CEF-CHL-GEN-PEN-SUL
0	Unpecified or Missing	Emfuleni	2012	*S. aureus*	AMP-CEF-CHL-GEN-PEN-SUL
0	Unpecified or Missing	Tshwane	2012	*S. aureus*	AMP-CEF-DOX-GEN-PEN-SUL
84	Thoroughbred	Ekurhuleni	2007	*S. aureus*	AMP-CEF-DOX-GEN-PEN-SUL
168	Europeanwarmblood	Ekurhuleni	2012	*S. aureus*	AMP-CEF-CHL-GEN-PEN-SUL
0	American Saddler	Ekurhuleni	2007	*S. aureus*	AMP-CEF-DOX-GEN-PEN-SUL
81	Thoroughbred	Tshwane	2009	*S. aureus*	AMP-CEF-DOX-GEN-PEN-SUL
0	American Saddler	Ekurhuleni	2007	*Staph. spp.*	AMP-CEF-DOX-GEN-PEN-SUL
48	Europeanwarmblood	Westonaria	2012	*S. aureus*	AMP-CHL-DOX-GEN-PEN-SUL
19	Europeanwarmblood	Westonaria	2012	*S. aureus*	AMP-CHL-DOX-ENR-GEN-PEN-SUL
108	Thoroughbred	Tshwane	2009	*S. aureus*	AMP-CEF-CHL-DOX-GEN-PEN-SUL
0	Arab	Johannesburg	2012	*S. aureus*	AMI-AMP-CHL-DOX-GEN-PEN-SUL
168	Europeanwarmblood	Johannesburg	2012	*S. aureus*	AMP-CEF-CHL-DOX-GEN-PEN-SUL
36	Europeanwarmblood	Westonaria	2010	*S.pseudintermedius*	AMP-CEF-CHL-DOX-ENR-GEN-SUL
111	All other breeds	Tshwane	2009	*S.pseudintermedius*	AMP-CEF-CHL-DOX-GEN-PEN-SUL
96	American Saddler	Tshwane	2012	*S.aureus*	AMI-AMP-CEF-CHL-GEN-PEN-SUL
0	Nooitgedachtpony	Tshwane	2009	*S. aureus*	AMI-AMP-CEF-CHL-DOX-ENR-GEN-PEN
120	Europeanwarmblood	Johannesburg	2009	*S.epidermidis*	AMI-AMP-CEF-DOX-ENR-GEN-PEN-SUL
182	Boerperd	Tshwane	2012	*S.aureus*	AMP-CEF-CHL-DOX-ENR-GEN-PEN-SUL
48	Europeanwarmblood	Tshwane	2012	*S.epidermidis*	AMP-CEF-CHL-DOX-ENR-GEN-PEN-SUL
155	Thoroughbred	Johannesburg	2012	*S.aureus*	AMI-AMP-CHL-DOX-ENR-GEN-PEN-SUL
83	Thoroughbred	Johannesburg	2011	*S.pseudintermedius*	AMI-AMP-CEF-CHL-DOX-ENR-GEN-PEN-SUL
0	Unpecified or Missing	Tshwane	2009	*S.aureus*	AMI-AMP-CEF-CHL-DOX-ENR-GEN-PEN-SUL
72	Thoroughbred	Tshwane	2009	*S.aureus*	AMI-AMP-CEF-CHL-DOX-ENR-GEN-PEN-SUL

^a^AMI = Amikacin; AMP = Ampicillin; CEF = Ceftriaxone; CHL = Chloramphenicol; DOX = Doxycycline; ENR = Enrofloxacin; GEN = Gentamicin; PEN = Penicillin; SUL = Sulphametho

## Discussion

The current study is an exploratory descriptive analysis of antimicrobial resistance patterns of *Staphylococcus* spp. isolated from horses presented at a veterinary teaching hospital in South Africa. Since very little is known regarding the epidemiology of antimicrobial resistance in horses in Africa and many other developing economies, this study is intended to provide preliminary information to guide future more detailed hypothesis driven epidemiological studies of antimicrobial drug resistance among *Staphylococcus* spp. not only in horses, but other domestic species as well.

Contrary to findings by Leekha et al. [30] the present study did not find significant seasonal differences in the percentage of *Staphylococcus* positive samples. This could be due to aggregation of cases by pre-defined seasons, as was done in the present study. Other authors have argued that this aggregation leads to loss of information if infection occurrences are not seasonal but follow other cyclical patterns such as biannual patterns [30]. Some authors have suggested use of time-series analytical approaches in such situations [31]. Unfortunately, due to the exploratory nature and the small samples sizes of the current study, time-series analysis could not be performed. The significant association observed between season and percentage of antimicrobial resistance could be attributed to weather conditions such as temperature and humidity. However, it may also be due to seasonal differences in staffing levels (i.e., fewer regular staff during vacation months) and seasonal differences in antimicrobial drug use due to differences in infection rates from other bacteria resulting in differences in selection pressure [30].

Although the percentages of *Staphylococcus* positive samples increased over time, there were no similar increases in the number of samples submitted for processing. Therefore, the observed increases in percentage of *Staphylococcus* positive samples may be a reflection of a true increase in the number of staphylococcal infections over the years. However, more detailed primary base studies will need to be performed to further investigate this trend. The higher levels of Staphylococcal infections in some breeds of horses seem to suggest potential breed predisposition. However, the reason for this is unclear and no previous studies have reported this. Therefore, this warrants further investigations to establish the potential existence of breed predisposition. Although sex dimorphism of staphylococcal infections, especially methicillin resistant infections, have been observed in humans [32], no similar associations were observed in this study and to our knowledge, no studies have reported differences in staphylococcal infections in horses based on sex.

Coagulase positive *Staphylococcus* spp., especially *S. aureus* are responsible for a significantly large percentage of infections in horses as opposed to coagulase negative strains [1]. Therefore, the higher levels of resistance observed among *S. aureus* in this study is of concern, from a therapeutic point of view, due to the resulting potential treatment failures associated with the observed high levels of resistance. The relatively high levels of AMR and MDR observed in this study may suggest high selection pressure among *Staphylococcus* spp. isolates from horses treated at the veterinary teaching hospital. This may be due to the fact that most of the cases seen in the teaching hospital tend to be referral cases that may not have responded well to initial antimicrobial treatments by the primary care veterinarians. Thus, it is possible that most of these horses would have been on antibiotic treatment for prolonged periods before being transferred to the referral veterinary teaching hospital. Another plausible explanation for the high levels of antimicrobial resistance observed in the study could be the result of failure of horse owners to ensure that the animals complete the full course of prescribed antimicrobial therapy. This issue has been highlighted by some authors who reported that caretakers have a tendency to stop drug administration as soon as disease symptoms abate, a practice that has been incriminated in the development of antimicrobial drug resistance [17]. Transportation stress has also been incriminated in the high carriage of resistant *Staphylococcus* in equine patients admitted to clinics as a result of direct contact with referring veterinarians who might be carrying resistant *Staphylococcus* [4].

The high levels of antimicrobial resistance observed in this study is consistent with the observations in humans in South Africa by Essa et al. [12], who observed that up to 95.1% of the samples were MDR, and only 3.7% of the samples were susceptible to all antibiotics tested in the study. The very high levels of resistance to β-lactams observed in the current study is consistent with reports by Weese [1] who reported high levels of resistance to β-lactams among staphylococci and especially *S. aureus*. Some studies have reported that routine use of β-lactam antibiotics in prevention of surgical infections, predisposes horses to acquisition of methicillin resistant *S. aureus* [33, 34]. Therefore, the high levels of resistance to β-lactam antibiotics in the present study could signal the existence of methicillin resistance in staphylococcal infections in horses presented at the hospital under study. Unfortunately, methicillin was not routinely included in the susceptibility test panels used by the veterinary teaching hospital that supplied the data for the current study, and hence the levels of resistance to methicillin could not be assessed in the current study. However, it is encouraging to note that resistance to fluoroquinolones was relatively low implying that these antimicrobials are still relatively effective and therefore their use could more likely be associated with successful treatment outcomes compared to β-lactams. It is worth noting that in South Africa, some of the older antibiotics are readily available to farmers over the counter while the newer ones require prescription [35]. This may have impacted selection pressure for antimicrobial resistance especially influencing antimicrobials readily available over the counter.

The observed low levels of resistance towards aminoglycosides was not unusual. For instance, Abrahamsen [17] in a study conducted in Maine (USA), also observed that *Staphylococcus* spp. tended to be susceptible to both gentamicin and amikacin, both of which are aminoglycosides. Furthermore, a study by Schnellmann et al. [33], investigating emergence of resistance after hospitalization in Switzerland, found much lower levels of resistance to amikacin compared to other antimicrobials. However, it is worth noting that the level of resistance observed here may not be a true reflection of the level of resistance in the larger horse population. For example, Van den Eede et al. [4] observed a much lower level of resistance among horses on farms that were situated in a region surrounding an equine clinic in Belgium whereas much higher carriage rates of resistant *Staphylococcus* had been detected in horses presented at the clinic [4].

The significant differences in the percentage of MDR isolates across species, with *S. aureus* exhibiting higher levels of MDR compared to other species, was anticipated given that it is the most common species in horses and therefore more likely to be exposed to higher selection pressure during antibiotic treatments compared to the other less common species [30]. Worth noting was that MDR was frequently observed among isolates from horses less than 1 month old. This could be due to the fact that foals frequently require antimicrobial treatment to combat a variety of conditions. Moreover, the basic principle of antimicrobial treatment in neonatal foals is the use of broad-spectrum antimicrobials [36]. This practice potentially increases the selection pressure for antimicrobial resistance among foals. However, it is interesting to note that MDR isolates resistant to six or more drugs tended to occur in older horses, which suggests that the overall selection pressure for antimicrobial drug resistance could be higher in older horses. Some authors have indicated that after 3 days of hospitalization and treatment with penicillin, the percentage of *Staphylococcus* isolates showing antibiotic resistance dramatically increases in horses [33].

Although some authors have reported geographic differences in levels of AMR staphylococcal isolates [37], the current study did not identify significant geographic differences. This could most likely be due to the small samples sizes involved in some of the municipalities included in the study. Therefore, more detailed and larger studies need to be performed to more fully investigate geographic and other determinants of variations in AMR and MDR staphylococcal infections. Thus, our next set of studies will focus on these issues as well as investigating practices in the use of antimicrobials by both veterinary practitioners and animal owners.

A limitation of this retrospective laboratory-based study is that the source of samples (e.g. nasal, skin, etc) could not be assessed due to quality of these data. A positive outcome of this finding is that we will work with the laboratory to improve laboratory data capture. This will have a positive impact of improved data quality for similar studies in the future. Additionally, information on past antimicrobial use was not available and therefore we could not assess its associations with levels of AMR or MDR. Moreover, detailed analysis of the β-lactam resistant isolates were not performed and hence we could not assess their susceptibility to methicillin. Finally, it is possible that some of the isolates reported as *Staphylococcus* spp. may have belonged to *S. aureus*, *S. pseudintermedius*, *S. epidermidis* or *S. equinus*. Unfortunately, information was not available to elucidate this.

## Conclusions

The above limitations notwithstanding, the study has shown evidence of seasonal patterns of staphylococcal infections and occurrence of AMR and MDR in horses. There is also evidence of differences in the occurrence of AMR and MDR by species of *Staphylococcus* and breed of horse. Resistance among *Staphylococcus* isolates was highest towards β-lactam antibiotics, but lowest towards amikacin. It should be pointed out that this was an exploratory descriptive study that did not lend itself to more detailed analyses such as use of multivariable models due to the small sample sizes involved. However, this being the first study of its kind in Africa, it provides useful descriptive information to guide future more detailed studies intended to address the problems of equine staphylococcal infections and antimicrobial resistance. Routine surveillance, prudent antimicrobial use and efficient infection control should be advocated as strategies to contain development of antimicrobial resistance in horse infections. Future studies will need to be primary-base involving large sample sizes to help understand specific local factors contributing to development of antimicrobial resistance so as to better guide treatment and control efforts.

## Abbreviations

95% CI: 95% Confidence Interval
AMR: Antimicrobial Resistant
CoNS: coagulase-negative staphylococci
CoPS: coagulase-positive staphylococci
MDR: Multidrug Resistant
MRSA: Methicillin Resistant *Staphylococcus aureus*
p: *p*-value
*S. aureus*: *Staphylococcus aureus*
*S. epidermidis*: *Staphylococcus epidermidis*
*S. equinus*: *Staphylococcus equinus*
*S. hyicus*: *Staphylococcus hyicus*
*S. intermedius*: *Staphylococcus intermedius*
*S. pseudintermedius*: *Staphylococcus pseudintermedius*
spp.: Species
USA: United States of America
β-lactam: Beta-lactam

## References

[1] Weese JS. Staphylococcal infections. In: Sellon DC, Long M, editors. Equine Infect Dis Internet. Toronto: Elsevier Health Sciences; 2013. p. 664.

[2] Devriese LA, Nzuambe D, Godard C (1985). Identification and characteristics of staphylococci isolated from lesions and normal skin of horses. Vet Microbiol.

[3] Busscher JF (2006). Van Duijkeren E, Sloet van Oldruitenborgh-Oosterbaan MM. The prevalence of methicillin-resistant staphylococci in healthy horses in the Netherlands. Vet. Microbiol.

[4] den Eede A, Martens A, Feryn I, Vanderhaeghen W, Lipinska U, Gasthuys F (2012). Low MRSA prevalence in horses at farm level. BMC Vet Res.

[5] van Duijkeren E, Ten Horn L, Wagenaar JA, de Bruijn M, Laarhoven L, Verstappen K (2011). Suspected horse-to-human transmission of MRSA ST398. Emerg Infect Dis [Internet].

[6] van Duijkeren E, Moleman M, van Oldruitenborgh-Oosterbaan MM, Multem J, Troelstra A, Fluit AC (2010). Methicillin-resistant Staphylococcus Aureus in horses and horse personnel: an investigation of several outbreaks. Vet Microbiol.

[7] Weese JS, Caldwell F, Willey BM, Kreiswirth BN, McGeer A, Rousseau J (2006). An outbreak of methicillin-resistant Staphylococcus Aureus skin infections resulting from horse to human transmission in a veterinary hospital. Vet Microbiol.

[8] Weese JS, Archambault M, Willey BM, Hearn P, Kreiswirth BN, Said-Salim B (2005). Methicillin-resistant Staphylococcus Aureus in horses and horse personnel, 2000-2002 141. EmergInfectDis Centers Dis Control Prev.

[9] Perovic O, Koornhof H, Black V, Moodley I, Duse A, Galpin J (2006). Staphylococcus Aureus bacteraemia at two academic hospitals in Johannesburg. South African Med J.

[10] Magiorakos A-P, Srinivasan A, Carey RB, Carmeli Y, Falagas ME, Giske CG, et al. Multidrug-resistant, extensively drug-resistant and pandrug-resistant bacteria: an international expert proposal for interim standard definitions for acquired resistance. Clin. Microbiol. Infect. [Internet]. Blackwell Publishing Ltd; 2012 [cited 2016 Jun 24];18:268–81 Available from: http://linkinghub.elsevier.com/retrieve/pii/S1198743X14616323 10.1111/j.1469-0691.2011.03570.x 21793988

[11] Safdar N, Bradley EA (2008). The risk of infection after nasal colonization with Staphylococcus Aureus. Am J Med [Internet].

[12] Essa ZI, Connolly C, Essack SY. *Staphylococcus aureus* from Public Hospitals in Kwa-Zulu-Natal, South Africa–Infection Detection and Strain-Typing. South African J. Epidemiol. Infect. [Internet]. 2009 [cited 2016 Jun 24];24:4–7 Available from: http://www.sajei.co.za/index.php/SAJEI/article/viewFile/100/143.

[13] Ruzauskas M, Couto N, Siugzdiniene R, Klimiene I, Virgailis M, Vaskeviciute L (2015). Methicillin-resistant coagulase-negative staphylococcus spp. prevalence in Lithuanian dogs: a cross-sectional study. Vet. Arh.

[14] Kern A, Perreten V (2013). Clinical and molecular features of methicillin-resistant, coagulase-negative staphylococci of pets and horses. J Antimicrob Chemother [Internet].

[15] Weese JS, Lefebvre SL (2007). Risk factors methicillin-resistant staphylococcus aureus colonization in horses admitted to a veterinary teaching hospital. Can Vet J.

[16] Bagcigil FA, Moodley A, Baptiste KE, Jensen VF, Guardabassi L (2007). Occurrence, species distribution, antimicrobial resistance and clonality of methicillin- and erythromycin-resistant staphylococci in the nasal cavity of domestic animals. Vet Microbiol.

[17] Abrahamsen K (2004). Equine infectious diseases and microbial resistance to antibiotics. Dartmouth Undergradaute J Sci..

[18] Oguttu WJ (2007). Antimicrobial drug resistance of enteric bacteria from broilers fed antimicrobial growth enhancers and exposed poultry abattior workers.

[19] Morton TM, Johnston JL, Patterson J, Archer GL. Characterization of a conjugative staphylococcal mupirocin resistance plasmid. DOI Antimicrob. Agents Chemother [Internet]. 1995 [cited 2017 Mar 4];39 Available from: http://aac.asm.org/content/39/6/1272. 10.1128/aac.39.6.1272 PMC162726 7574515

[20] Bjorland J, Steinum T, Sunde M, Waage S, Heir E. Novel plasmid-borne gene qacJ mediates resistance to quaternary ammonium compounds in equine *Staphylococcus aureus*, Staphylococcus simulans, and Staphylococcus intermedius. Antimicrob. Agents Chemother. [Internet]. 2003 [cited 2017 Mar 4];47:3046–52 Available from: http://aac.asm.org/cgi/doi/10.1128/AAC.47.10.3046-3052.2003. 10.1128/AAC.47.10.3046-3052.2003 PMC201118 14506007

[21] Moodley A, Oosthuysen WF, Duse AG, Marais E, South African MSG (2010). Molecular characterization of clinical methicillin-resistant Staphylococcus Aureus isolates in South Africa. J Clin Microbiol [Internet].

[22] Oosthuysen WF, Orth H, Lombard C, Sinha B, Wasserman E. In vitro characterization of representative clinical South African *Staphylococcus aureus* isolates from various clonal lineages. New microbes new Infect. [Internet]. Elsevier; 2014 [cited 2016 Jun 24];2:115–22. Available from: http://www.ncbi.nlm.nih.gov/pubmed/25356356. 10.1002/nmi2.53 PMC4184580 25356356

[23] SOUTHAFRICA.COM. Weather of Gauteng | By South Africa Channel [Internet]. South Africa Channels. 2017 [cited 2017 Mar 3] Available from: http://www.southafrica.com/gauteng/climate/.

[24] World Weather Online. Weather Averages | Monthly Average High and Low Temperature | Average Precipitation and Rainfall days | World Weather Online [Internet]. 2012 [cited 2017 Mar 3]. Available from: https://www.worldweatheronline.com/johannesburg-weather-averages/gauteng/za.aspx.

[25] Quinn PJ, Carter ME, Markey B, Carter GR (1994). Clinical veterinary microbiology.

[26] Clinical and Laboratory Standards Institute (2008). Performance standards for antimicrobial susceptibility testing; eighteenth informational supplement. CLSI docum.

[27] Dohne S, Merle R, Altrock AV, Waldmann KH, Verspohl J, Gruning P (2012). Antibiotic susceptibility of salmonella, campylobacter coli, and campylobacter jejuni isolated from northern German fattening pigs. J Food Prot.

[28] Royston P (1983). A simple method for evaluating the Shapiro-Francia W’ test for non-normality. Underst Stat.

[29] Corporation STATA (2013). Stata MP. Parallel E. 4905 Lakeway drive College Station.

[30] Leekha S, Diekema DJ, Perencevich EN (2012). Seasonality of staphylococcal infections. Clin Microbiol Infect [Internet].

[31] Fisman DN. Seasonality of Infectious Diseases. https://doi.org/10.1146/annurev.publhealth.28.021406.144128. Annual Reviews; 2007; 10.1146/annurev.publhealth.28.021406.144128 17222079

[32] Humphreys H, Fitzpatick F, Harvey, Brain J. Gender differences in rates of carriage and bloodstream infection caused by methicillin-resistant *Staphylococcus aureus*. Are they real, do they matter and why? Clin. Infect. Dis. [Internet]. 2015;61:1708–14. Available from: http://cid.oxfordjournals.org/content/early/2015/07/21/cid.civ576.full.pdf+html. 10.1093/cid/civ576 26202769

[33] Schnellmann C, Gerber V, Rossano A, Jaquier V, Panchaud Y, Doherr MG, et al. Presence of New mecA and mph(C) Variants Conferring Antibiotic Resistance in Staphylococcus spp. Isolated from the Skin of Horses before and after Clinic Admission. J. Clin. Microbiol. [Internet]. American Society for Microbiology; 2006 [cited 2016 Jun 24];44:4444–54 Available from: http://jcm.asm.org/cgi/doi/10.1128/JCM.00868-06 10.1128/JCM.00868-06 PMC1698435 17005735

[34] Yah SC, Enabulele I, Eghafona NO. The screening of multi-drug resistance (MDR) susceptibilities of *Staphylococcus aureus* and *Staphylococcus epidermidis* to methicillin and vancomycin in teaching hospitals in Nigeria. Sudan J. Med. Sci. [Internet]. Omdurman Islamic University; 2008 [cited 2016 Jun 24];2:257–62 Available from: http://www.ajol.info/index.php/sjms/article/view/38497.

[35] Henton MM, Eagar HA, Swan GE, van Vuuren M. Part VI. Antibiotic management and resistance in livestock production. S. Afr. Med. J. [Internet]. 2011 [cited 2017 Jul 15];101:583–6. Available from: http://www.ncbi.nlm.nih.gov/pubmed/21920137. 21920137

[36] Furr Martin O, Mogg T (2003). Antimicrobial treatment of neonatal foals. Compedium.

[37] Burton S, Reid-Smith R, McClure JT, Weese JS. *Staphylococcus aureus* colonization in healthy horses in Atlantic Canada. Can Vet J. 2008;49:797–9. PMC2465786 18978975

